# Conical Extremes of a Multivariate Sample

**DOI:** 10.6028/jres.099.049

**Published:** 1994

**Authors:** Alexander V. Gnedin

**Affiliations:** University of Göttingen, Göttingen, Germany

**Keywords:** conical extremes, multivariate extremes, regular variation

## Abstract

We introduce multivariate extremes in the direction of a given cone. Convergence results for the number of the *k*th extremes are obtained for sampling from a distribution having asymptotically independent radial and spherical components and regularly varying tail of the radial component.

## 1. Introduction

Let *ℋ_n_* = {*X*_1_,* …*, *X_n_*} be a point set of independent identically distributed *d*-dimensional random vectors sampled from the probability measure *μ*, and *K* be a punctured at the origin *cone* in **R***^d^*, *d* > 1. We define *the kth layer* as

$${ℒ}^{\left(k\right)}\left({ℋ}_{n}\right)=\left\{X_{i};\#\left(K_{x_{1}}\cap {ℋ}_{n}\right)=k-1\right\}\;k-1,2,\ldots ,$$

where *K_x_ = x+K* is the translated cone with vertex in *x* ∊ **R***^d^*. Intuitively, the *k*th layer is the set of the *k*th extremes of ℋ*_R_* in the direction *K.* The prime examples we have in mind are (1) *the Pareto-optimal points* corresponding to the first layer in the direction of the positive orthant, and (2) *the total maximum*, which may be considered as the first layer in the direction of the cone, complement to the negative orthant. We are interested here in the distributions of random variables

$$V_{n}^{\left(k\right)}=\#{ℒ}^{\left(k\right)}\left({ℋ}_{n}\right),$$

counting the number of points in the *k*th layer. These distributions depend essentially on both *K* and *μ*.

From a more general viewpoint, the first layer can be regarded as the set *of maximal elements* [4] with respect to the binary relation ℛ in **R***^d^* defined as *x*ℛ*y* ⇔ *x−y* ∊ *K*. Alternatively, any scale and translation invariant binary relation generates a cone by setting K = {*x* **∈** *R^d^*:*x*ℛ0} and the maximal elements are conical extremes.

Two above cases of the counting problem have been considered in the literature under the assumption that *μ* is either a product of one-dimensional marginal measures or a multivariate normal distribution (2,10,11,12]. It is well known, for example, that if *μ* is a product measure in **R***^d^* then the average number of Pareto points is of the order of (log *n*)*^d^*^−1^, while the probability that the multiple maximum exists is *n*^d−1^.

In this paper we focus on a class of distributions *μ* already studied in connection with multivariate extreme-value theory [8] and statistics of convex hulls [1,5,6,9]. These distributions are characterized by regular variation of the tail of the radial component and asymptotical independence of radial and angular components. We show that typically the *V_n_*^(^*^k^*^)^’*s* converge in distribution and the expectations have finite limits as *n* → ∞. In the special case of slow variation wc calculate explicitly the limiting distributions.

## 2. Preliminaries

We define a *cone* as a punctured at the origin, scale-invariant Borel set in **R***^d^*, i.e., 0 ∉ *K, tK = K* ∀*t* > 0. Each cone is uniquely determined by its spherical base *S*_+_= *K* ∩ *S*, where *S* denotes the unit sphere. We associate with *K* also the spherical set 5. obtained by reflection about the origin, *S*_0_ *− S\* (S_+_ ∪ *S*_−_) and *S*_2_ *= S_+_ ∩ S−.* The cone with spherical base *C ⊂ S* will be denoted cone (C).

Set $B_{r}=\left\{x\in R^{d}:\left\|x\right\|\leq r\right\}$, $B_{r}^{c}=R^{d}\backslash B_{r}$ and $A_{r,C}=\mathrm{cone}\left(C\right)\cap B_{r}^{c}$.

We fix in what follows a cone A and a multidimensional probability distribution *μ* satisfying the following conditions:

- There exists *a* ≥ 0 and a probability measure *ρ* on *S* such that
  $$\underset{t\to \infty }{\lim}\frac{\mu \left(B_{lr}^{c}\right)}{\mu \left(B_{l}^{c}\right)}=r^{-u}\;r>0,$$ (1)
- For all *ρ*-continuous *C* ⊂ *S*
  $$\underset{r\to \infty }{\lim}\frac{\mu \left(A_{r,C}\right)}{\mu \left(B_{r}^{c}\right)}=\rho \left(C\right),$$ (2)
- *ρ* (int *S*_+_) > 0, and
- *μ*. has no atom at the origin.

Consider an *iid* sample from *μ*., *H_n_ −* {*X*_1_ *…*, *X_n_*}, represented in the polar form as the product of radial and spherical components: *X_i_ = R_i_Z_i_*, where *R_i_* = ‖*X_i_*‖, *Z_i_* = *X_i_*/‖*X_i_*‖. The above conditions on *μ* have a natural probabilistic interpretation. Condition (i) means that the distribution function of the radial component,

$$F\left(r\right)\overset{\mathrm{def}}{=}\;\mu \left(B_{r}\right).$$

has a regularly varying tail. Condition (ii) is translated as

$$\underset{r\to \infty }{\lim}\;P\left\{Z_{1}\in \cdot |R_{J}>r\right\}=\rho \left(\cdot \right)$$

and is to be interpreted as the asymptotic independence of radial and spherical components, where the limiting distribution *ρ* does not disappear in the interior of *S_+_.* (condition (iii)). The last condition is not essential and assumed for technical reasons.

Given a Borei set *B ⊂* **R***^d^*, we represent the number of the *k*th layer points in *B* as the sum of random indicators

$$\#{ℒ}^{\left(1\right)}\left({ℋ}_{n}\cap B\right)=\sum_{j-1}^{n}1_{\left(X_{1}\in {ℒ}^{\left(1\right)}\left({ℋ}_{n}\right)\cap B\right)},$$

and using the *iid* assumption write for the expectations

$$\begin{array}{l} E\#{ℒ}^{\left(k\right)}\left({ℋ}_{n}\cap B\right)=nP\left\{X_{1}\in {ℒ}^{\left(k\right)}\left(ℋn\right)\cap B\right\}=\\ n\left(\begin{array}{c} n-1\\ k-1 \end{array}\right)P\left\{X_{1}\in B;X_{2},\ldots ,X_{k}\in K_{x_{1}};X_{k+1},\ldots ,X_{k}\notin K_{x_{1}}\right\}=\\ n\left(\begin{array}{c} n-1\\ k-1 \end{array}\right)\int_{0}{\left(\mu \left(k_{x}\right)\right)}^{k-1}{\left(1-\mu \left(K_{x}\right)\right)}^{n-k}d\mu \left(x\right). \end{array}$$ (3)

The following lemmas will be used to estimate these integrals.

**Lemma 1**. *There exists* τ > 0 *such that μ*(*K_x_*) >.τ(1−*μ*(B_‖x‖_)) *for all x* ∈ **R***^4^*

*Proof.* Consider first the case where there exists a linear isomorphism which maps *K* onto the positive orthant Let *y* be the inverse image of the vector (1, …,1) under this isomorphism. By convexity, *K_y_⊂ K_x_* for *x ∈ B*_1_.

Condition (iii) allows one to select a compact *ρ*-continuous set CC *S_+_* with ρ (*C*)>0. It is easy to see that *y* ∈ int *K*, the sets *K_sy_*, s>0, are increasing as *s ↓* 0 and ∪*_s_*_>0_ *K_sy_* = int *K.* It follows that *C* ⊂ *K_sy_* for sufficiently small s. Furthermore, for small *s* we have also *A*_1,_*_C_* ⊂ *K_sy_.* Indeed, the points of *A*_1,_*_c:_* are representable as *tx*, with *t* > 1, *x* ∈ *C*, thus, by convexity, *x* ∈ *K_sv_* implies *tx* ∈ *K_isy_ ⊂ K_sy_.* Homogeneity implies A*_t/s,C_* ⊂ *K_ry_.* It follows now from Eqs. (1) and (2) that

$$\begin{array}{r} \frac{\mu \left(K_{ry}\right)}{\mu \left(B_{r}^{c}\right)}>\frac{\mu \left(A_{r/s}C\right)}{\mu \left(B_{r}^{c}\right)}=\frac{\mu \left(A_{r/s}c\right)}{\mu \left(B_{r/s}^{c}\right)}\frac{\mu \left(B_{r/s}^{s}\right)}{\mu \left(B_{r}^{c}\right)}\\ \to s^{a}\rho \left(C\right).\;r\to \infty . \end{array}$$ (4)

From *K_x_* ⊃ *K_‖x‖_*, we derive for sufficiently large *r*_0_ and *‖x‖* > *r*_0_ that

$$\frac{\mu \left(K_{x}\right)}{\mu \left(B_{\left\|x\right\|}^{c}\right)}\geq \frac{\mu \left(K_{\left\|x\right\|y}\right)}{\mu \left(B_{\left\|x\right\|}^{c}\right)}>\frac{1}{2}s^{\alpha }\rho \left(C\right).$$

For $x\in B_{r_{0}}$ we have $K_{x}⊃K_{r_{0}y}$, therefore Eq. (4) along with the inclusion $K_{tr_{0}y}⊃K_{r_{0}y},\;t>1$, implies

$$\frac{\mu \left(K_{x}\right)}{\mu \left(B_{\left\|x\right\|}^{c}\right)}\geq \frac{\mu \left(K_{r_{o}y}\right)}{\mu \left(B_{\left\|x\right\|}^{c}\right)}>\mu \left(K_{r_{o}y}\right)>0.$$

The assertion follows in this case by setting $\tau =\min\left(\mu \left((K_{r\;0\;y}\right),\frac{1}{2}s^{\alpha }\rho \left(C\right)\right)$.

For arbitrary *K* one can find a smaller cone *K*′ ⊂ *K*, which is linearly isomorphic to the positive orthant and still has the interior of its spherical base of positive p-measure. This is possible since the spherical *d*-sim-plexes build a measure-generating class on *S.* It remains to note that $\mu \left(k_{x}\right)\geq \mu \left(K_{x}^{l}\right)$ for any translation, whence the estimate holds in general.☐

**Lemma 2.***If***E** V*_n_*^(1)^ *has a limiting value v* ∈ [0,∞) *then all EV_n_*^(^*^k^*^)^, *k* − 2,3,… *converge to this limit as n* → ∞

*Proof.* Let *m*(*t*). *t* ∈ [0,1], be the distribution function of the image measure induced by the mapping *X↦μ*(*K_x_*). Changing variables transform Eq. (3) to the one-dimensional Lebesgue-Stiltjes integral

$$EV_{n}^{\left(k\right)}=n\left(\begin{array}{c} n-1\\ k-1 \end{array}\right)\int_{0}^{1}t^{k-t}{\left(1-t\right)}^{n-k}dm\left(t\right).$$

*A* slight modification of the standard Tauberian theorem as found in [14] assures that the limiting value of this integral for *k* = 1 exists iff *m*(*t*) is left-differentiable at *r* = 1, in which case the limit and the derivative have the same value. Applying this theorem in the reverse direction one can easily see that all the **E***V_n_*^(^*^k^*^)^ ‘s must have the same limit. □

**Lemma 3.***Assume γ_n_. is an increasing sequence such that*$\underset{n\to \infty }{\lim}\;n\left(1-F\left(\gamma_{\alpha }\right)\right)\to \gamma ,\gamma >0$, *then*

$$\underset{n\to \infty }{\lim\;\sup}\;{\mathrm{EV}}_{n}^{\left(k\right)}⩽\tau^{-1},$$ (5)

$$\underset{n\to \infty }{\lim\;\sup}E\;\#\;\left({ℒ}^{\left(1\right)}\left({ℋ}_{n}\right)\cap B_{\gamma n}\right)⩽e^{\gamma \tau }\tau^{-1},$$ (6)

*with* τ *determined by Lemma* 1.

*Proof.* Set

$$f\left(t\right)=\int_{F\left(r\right)⩽t}dF\left(r\right),t\in \left[0,1\right].$$

Regular variation of *F* at infinity implies readily that for all sufficiently distant discontinuity points the ratio (jump-size)/(distribution tail) is close to zero. It follows that (1-*f*(*t*))/(1−*t*)→1 as *t* ↑ 1 (for continuous *F* this is obvious since *f*(*t*) = *t*). Lemma 1, a change of variables and the Tauberian theorem yield

$$\begin{array}{c} EV_{n}^{\left(1\right)}=n\int_{R^{d}}{\left(1-\mu \left(K_{x}\right)\right)}^{n-1}d\mu \left(x\right)⩽\\ n\int_{R^{d}}(1-\tau {\left(1-\mu \left(B_{\left\|x\right\|}\right)\right)}^{n-1}d\mu \left(x\right)=\\ \int_{R}(1-\tau {\left(1-F\left(r\right)\right)}^{n-1}dF\left(r\right)=\int_{0}^{1}{\left(1-\tau \left(1-t\right)\right)}^{n-1}df\left(r\right)\to \tau^{-1}. \end{array}$$

Similarly,

$$\begin{array}{l} \underset{n\to \infty }{\lim\;\sup}\;E\#\left({ℒ}^{\left(1\right)}\left({ℋ}_{n}\right)\cap B\gamma_{n}\right)⩽n\int_{0}^{\gamma_{n}}(1-\tau {\left(1-F\left(r\right)\right)}^{n-1}\\ dF\left(r\right)=n\int_{0}^{F\left(\gamma_{n}\right)}{\left(1-\tau \left(1-t\right)\right)}^{n-1}df\left(t\right)\sim n\int_{0}^{\gamma }{\left(1-\tau \left(1-t\right)\right)}^{n-1}dt\to e{\;}^{\gamma }\tau^{-1}, \end{array}$$

where the equivalence can be justified by partial integration. ☐

## 3. Pareto-Tails: *α* > 0

In this section we study the limiting behaviour of *V_n_*^(^*^k^*^)^ under the assumption that the regular variation index *α* in Eq. (1) is positive. Our plan is to translate Eqs. (1) and (2) into the convergence, of a suitably normalized sample, to a Poisson process[6, 15] and then apply a continuity argument to prove also the convergence of the *V_n_*^(^*^k^*^)’^S.

Compactify **R***^d^* by adjoining the infinite point *∞* and then puncture in the origin. The resulting topological space, say ${\hat{R}}^{d}$, is isomorphic to **R***^d^* and canonically embedded into its compactification, bounded from the origin Borel sets $B\subset {\hat{R}}^{d}$ being relatively compact. Wc endow the space $M\left({\hat{R}}^{d}\right)$ of Radon measures with the vague topology: $m_{n}\;\overset{v}{\to }\;m\;\mathrm{iff}\;m_{n}\;\left(B\right)\to m\left(B\right)$ for all relative compacts.

There exists a sequence of positive constants *a_n_*→∞ such that the measures $\nu_{n}\left(\cdot \right)\overset{\mathrm{def}}{=}n\mu \left(a_{n}\cdot \right)$ converge vaguely to the measure *v* determined by

$$v\left(A_{n}C\right)=r^{-\alpha }\rho \left(C\right),\;v\left(\left\{\infty \right\}\right)=0.$$ (7)

The limiting measure is in $M\left({\hat{R}}^{d}\right)$, being infinite on balls centered at the origin as well as on the sets cone (*C*) with *ρ*(*C*) > 0. In particular, condition (iii) implies *v* (int *K*) = *∞.* Clearly, *v* is a product measure in polar coordinates and has no atoms.

Let *ξ* be a Poisson point process in ${\hat{R}}^{d}$ with intensity measure *v*, and ξ*_n_*, be the random element of $M\left({\hat{R}}^{d}\right)$ associated with the scaled sample $a_{n}^{-1}{ℋ}_{n}$. Obviously, the operation of taking a layer commutes with rescaling: *ℒ*^(^*^k^*^)^ (*a*ℋ*_n_*) *− aℒ*^(^*^k^*^)^ (ℋ*_n_*), *a* > 0, therefore the number of points in each layer remains invariant under scale transformations. One can expect in this situation that *V_n_*^(^*^k^*^)^ converges in some sense to an analogous functional of the Poisson process.

Define the *k*th layer of the Poisson sample as

$${ℒ}^{\left(k\right)}\left(\xi \right)=\left\{x\in {\hat{R}}^{d}:\xi \left(\left\{x\right\}\right)=1,\;\xi \left(K_{x}\right)=k-1\right\}.$$

and denote *V*^(^*^k^*^)^ = #*ℒ*^(^*^k^*^)^(*ξ*) the number of points in the *k*th layer.

Using the polar representation, *x* = *rz*, and homogencity we can write *v(K_z_) = r^−α^ϕ*(*z*), where ϕ(*z*) = *v*(*K*) is a function of the spherical argument, Using Palm probabilities and integrating along radial rays we represent the expectations as

$$\begin{array}{l} EV^{\left(k\right)}=\int_{{\hat{R}}^{d}}e{\;}^{v}\left(k_{1}\right)\frac{{\left(v\left(K_{x}\right)\right)}^{k-1}}{\left(K-1\right)!}\;dv\left(x\right)=\\ \int_{S}\int_{R_{+}}c^{-r}{{\;}^{a}}^{\phi \left(z\right)}\frac{{\left(r^{-a}\phi \left(z\right)\right)}^{k-1}}{\left(k-1\right)1}dp\left(z\right)d\left(z\right)d\left(-r{\;}^{a}\right)=\\ \int_{S}\frac{d\rho \left(z\right)}{\phi \left(z\right)}. \end{array}$$ (8)

The resulting integral does not depend on *k*, as it is suggested by Lemma 2. The integration area can be reduced to *S*\int *S* since *ϕ* is infinite on int *S_*.

The following lemma is found in [5].

**Lemma 4.***Let E be a locally compact, Hausdorff and separable space; h*_0_,* h*_1_,*… be a uniformly bounded sequence of real measurable fittici ions commonly supported by a relatively compact set; and m*_0_, *m*_1_,… *be a sequence of Radon measures on E such that* $m_{n}\overset{v}{\to }m_{o}$. *The set D* = {*x* **∈** *E* : ∃{*x_n_*}, *x_n_→x, h_n_*(*x_n_*) *↛ h*(*x*)} *is measurable and if m*_o_(*D*)= 0 *then* ∫ *h_n_dm_n_* →*∫ h*_o_*dm*_o_.

Now we are ready to prove a convergence result.

**Theorem 1.***Assume* (i)–(iv), *α > 0, and*

v(−∂K)=0, (9)

$$v\times v\left\{\left(x,y\right)\in {\hat{R}}^{d}\times {\hat{R}}^{d}:\left(x-y\right)\in \partial K\right\}=0.$$ (10)

*Then for all k* = 1,2,…

$$\left(V_{n}^{\left(1\right)},\ldots ,V_{n}^{\left(k\right)}\right)\overset{d}{\to }\left(V^{\left(1\right)},\ldots V^{\left(k\right)}\right),$$ (11)

$$EV_{n}^{\left(k\right)}\to EV^{\left(k\right)},n\to \infty .$$ (12)

*Proof.* By Skorohod’s theorem we can find random point measures ${\hat{\xi }}_{n}\;,\;\xi \;\in \;M\left({\hat{R}}^{d}\right)$ satisfying ${\hat{\xi }}_{n}\overset{d}{=}\xi_{n}$, $\hat{\xi }\overset{d}{=\xi }$ and ${\hat{\xi }}_{n}\overset{v}{\to \hat{\xi }}$ a.s. Thus to prove the convergence in distribution (10) it suffices to consider the case $\xi_{n}\overset{v}{\to }\xi$ a.s. In what follows wc fix a typical realization of *ξ* and assume *n* sufficiently large.

Since *v*(int *K*)*= ∞, ξ* lays in the cone interior infinitely many points. Select *k* of them, say *x*_1_,…, *x_k_*, Pick *r* sufficiently small to satisfy $B_{r}\subset \;\cap_{j-1}^{k}\;-K_{x_{J}}$ as well as ξ(*∂*B_r_) = 0 and also *B_r_* ∩ {*x*_1_, …, *x_k_*} = 0. The complement $B_{r}^{c}$ is relatively compact hence the processes *ξ_n_* and *ξ* have there a finite number of points, say *y_n_*_.1_,…, *y_n.p_* and *y*_1_,… *y_p_*, respectively. These points may be labeled so that *y_n_*_.1_ → *y*_1_, as it follows from the vague convergence. By the construction, any translated cone *K_x_* with *x* ∈ *B_r_* contains at least *k* points of *ξ*, thus *B_r_* ∩ ℒ^(^*^j^*^)^(ξ) = 0 and also *B_r_* ∩ ℒ^(^*^j^*^)^(*ξ_n_*) = 0 for j = 1,…, *k.*

For *y*_i_ *∈* -int *K*, the cone *K_yi_* contains a vicinity of the origin, where ξ has infinitely many points. Therefore $y_{i}\notin \;\cap_{j-1}^{k}\;{ℒ}^{\left(k\right)}\left(\xi_{n}\right)$.

The condition shown in Eq. (9) assures that no one of *y*_1_,*…, y_p_* lies on *−∂K.* almost surely.

For *y_i_* ∉−cl *K*, the shifted cone $K_{y_{i}}$ is bounded from the origin. Therefore there exists an open vicinity of $\mathrm{cl}\left(\cup_{i}^{p}=1\;K_{y_{r}}\right)$ which is still relatively compact, and hence contains at most a finite number of points in addition to *y*_1_ …, *y_p_*. By Eq. (10), $\xi \left(\partial K_{y_{1}}\right)=0$ a.s. Again the poinwise convergence implies $\xi \;\left(K_{y_{n1}}\right)=\xi \left(K_{y1}\right)$, whence (*V_n_*^(1)^,…,*V_n_*^(^*^k^*^)^) = (*V*^(1)^, …,*V*^(^*^k^*^)^) and thus (11).

Now turn to the convergence in mean. It is enough to prove Eq. (12) for the first layer, *k* = 1. It is easy to see that

$$\begin{array}{l} E{V_{n}}^{\left(1\right)}\sim \int \exp\left(-v_{n}\left(K_{x}\right)\right)dv_{n}\left(x\right)=\int_{B_{r}}\left(\ldots \right)dv_{n}+\\ \int_{B_{r}^{c}}\left(\ldots \right)dv_{n}\;r>0 \end{array}$$

Take a point *x* ∉ −cl *K* and a sequence *x_n_* → *x* and consider the indicator functions of the sets *K_x_* and $K_{x_{n}}$ as the *h’s* in Lemma 4. The divergence set *D* is *∂K_xr_*, whence by (9) and the lemma $v_{n}\;\left(K_{x_{a}}\right)\to v\left(K_{x}\right)$.

For *x* ∈ −int *K*, *x_n_→x* we have $v_{n}\left(K_{x_{n}}\right)\;\mathrm{to}\;v\left(K_{1}\right)=\infty$ since $K_{x_{n}}$ contains some fixed vicinity of the origin for all sufficiently large *n.* Therefore in this case also $v_{n}\left(K_{x_{n}}\right)\to v\left(K_{x}\right)=\infty$.

To make further use of Lemma 4, consider this lime the functions *h*_0_(*x*) = exp(*−v*(*K_s_*)). $h_{n}\left(x\right)=\exp\left(-v\left(K_{xn}\right)\right)$. For the discontinuity set we have *D* ⊂ *−∂K* ∪ {*x : v*(*∂K_s_*) > 0}. The assumptions in Eqs. (9) and (10) imply *v*(*D*) = 0, hence for any *r*

$$\int_{n_{r}^{c}}\exp\left(-v_{n}\left(K_{x}\right)\right)dv_{n}\left(x\right)\to \int_{B_{r}^{c}}\exp\left(-v\left(K_{x}\right)\right)dv\left(x\right).$$

Now apply Lemma 3 to derive the estimate

$$\underset{n\to \infty }{\lim\;\;\sup}\int_{R_{1}}\exp\left(-v_{n}\left(K_{x}\right)\right)dv_{n}\left(x\right)⩽\tau {\;}^{1}\exp\left(-v\left(B_{r}^{c}\right)\tau \right).$$

The right-hand side here tends to zero as *r*→0.

Putting this all together and comparing with Eq. (8) we conclude

$$\underset{n\to \infty }{\lim\;\sup}EV_{n}^{\left(1\right)}⩽EV^{\left(1\right)}.$$

The reverse inequality,

$$\underset{n\to \infty }{\lim\;\inf}E{V_{n}}^{\left(1\right)}⩾EV^{\left(1\right)},$$

follows from the convergence in distribution □

**Remark.** The continuity conditions of Eqs, (9) and (10) are actually some properties of the spherical measure *p.* The first one trivially translates as *ρ* (*−∂K*) = 0, but we have not been able to find a re-formulation for the second. Sufficient conditions for Eq. (10) are: *p* is non-singular, and *∂*S_+_. lies in a (*n*−2)-dimcnsional set; or *K* is convex, *∂K* has no two-dimensional facets and *ρ*(*∂S*) =0.

**Example.** Here is a remarkable case where the expectations are explicitly computed. Consider the two-dimensional Cauchy distribution specified by the density d*μ*(*x*) = (2*π*)^−1^(1+‖*x*‖)^−3/2^d*x*. *x* ∈ **R**^2^. The radial tail is regularly varying with *α* = 1 and the circular measure is uniform, i.e., *dv* (*rz*) = (2π)^−1^*r* ^2^d*r* d*z*, *r* > 0, *z* ∈ [0, 2*π*).

Assume first that *K* is the positive quadrant The *k*th layer arc those *X_i_*’s which are exceeded by exactly *k* − 1 points of *ℋ_n_* in both components. Integrating yields

$$\phi \left(z\right)=v\left(K_{2}\right)=\frac{\cos\;z\;+\sin\;z-1}{2\pi \;\sin\;z\;\cos\;z}\;z\in \left(-\pi /2,\;\pi \right)$$

and *ϕ*(*z*) = ∞ otherwise. Computing the integral in Eq. (8) we obtain

$$\underset{n\to \infty }{\lim}\;EV_{n}^{\left(k\right)}=1+\frac{3\pi }{4}.$$

For *k* = 1 we have the limiting mean of the number of Pareto points.

Now suppose *K* is the complement to the negative quadrant. The *k*th layer consists of those *X_i_’s* which exceed all except some *k*−1 sample points in both components. We get

$$\phi \left(z\right)=\frac{\cos\;z+sin\;z+1}{2\pi \;\sin\;z\;\cos\;z}z\in \left(0,\pi /2\right)$$

and *ϕ*(*z*) = ∝ otherwise. Computing the integral Eq. (8) in this case, we obtain

$$\underset{n\to \infty }{\lim}\;{EV}_{n}^{\left(k\right)}=1-\frac{\pi }{4}.$$

The first layer is either empty or just one point, maximizing both components, thus this mean value coincides with the limiting probability of the total maximum.

The limiting distribution and higher moments of the $V_{n}^{\left(k\right)}$ can be, in principle, expressed in terms of some integrals similar to Eq. (8). These expressions do not seem tractable by analytical methods because of the complicated integration domains.

## 4. Slowly Varying Tails: *α* = 0

The case of slowly varying radial tail, with *α* = 0 in Eq. (1), is of special interest. The above Poisson approximation method does not work, since the sample cannot be rescaled to provide a non-degenerate limit. To get around, we extend here a method already exploited in [1], where the number of convex hull extremes of a sample under slighly stronger assumptions on the distribution has been studied.

We assume for technical reasons that *F* is continuous though, in fact, slow variation is all that is needed.

Let $X_{n}^{\left[1\right]},\ldots ,X_{n}^{\left[n\right]}$ be the elements of *ℋ_n_* arranged in the norm-decreasing order, i.e., $||X_{n}^{\left[1\right]}||\;>\;\ldots >\;||X_{n}^{\left[n\right]}$. Set $R_{n}^{\left|s\right|}=R_{j}$ and $Z_{n}^{\left|i\right|}=Z_{j}$, iff $X_{n}^{\left[i\right]}=X_{j};\;i,j=1,\ldots ,n$. One can recognize in the $R_{n}^{\left[i\right]}$ ’s the radial order statistics. The associated spherical variables, $Z_{n}^{\left[i\right]}$ ‘s will be called *concomitants.* Note that the continuity hypothesis make the definitions correct since the radial components are different with probability one.

Maller and Resnick [13] proved that slow variation is equivalent to

$$\frac{R_{n}^{\left[i+1\right]}}{R_{n}^{\left[j\right]}}\overset{v}{\to }0\;i=1,2,.\ldots$$ (13)

Our convergence results effectively exploit this fact combined with the asymptotic independence of the concomitants shown next.

Let Z^[1]^, Z^[2]^,… be iid S-valued random variables with distribution *ρ*.

**Lemma 5.***Assume that F*(*r*) = *μ*(*B_r_*) *is continuous and*Eq. (2)*holds. Then*

$$\left(Z_{n}^{\left[1\right]},\ldots ,Z_{n}^{\left[k\right]}\right)\overset{d}{\to }\left(Z^{\left[1\right]},\ldots ,Z^{\left[k\right]}\right)\;k=1,2,\ldots$$

*Proof.* For *ρ*-continuous *CCS* write (2) as

$$\underset{\gamma \to \infty }{\lim}\;\frac{1-F_{c}\left(r\right)}{1-F\left(r\right)}=\rho \left(C\right),$$ (14)

where *F_C_(r) = μ*(cone (*C*) ∩ *B_r_*). Select arbitrary *k* ∈ N and *ρ*-continuous spherical sets *C*_1_,…, *C_k_*.

We have

$$\begin{array}{c} P\left\{Z_{n}^{\left[1\right]}\in C_{1},\ldots ,Z_{n}^{\left[k\right]}\in C_{k}\right\}=\frac{n!}{\left(n-k\right)!}\\ P\{Z_{n}^{\left[1\right]}\in C_{1},X_{n}^{\left[1\right]}=X_{1},\ldots ,Z_{n}^{\left[k\right]}\in C_{k},X_{n}^{\left[k\right]}=\\ X_{k}\}=\frac{n!}{\left(n-k\right)!}P\{Z_{a}^{\left[1\right]}\in C_{1},R_{n}^{\left[1\right]}=R_{1},\ldots ,\\ Z_{n}^{\left[k\right]}\in C_{k},R_{n}^{\left[k\right]}=R_{1}\}=\frac{n!}{\left(n-k\right)!}P\{Z_{n}^{\left[1\right]}\in C_{1},\ldots ,\\ Z_{n}^{\left[k\right]}\in C_{k},R_{1}>\ldots >R_{k};R_{k}>R_{i}\;\text{for}\;i=k+J,\ldots ,n\}=\\ \frac{n!}{\left(n-k\right)!}\int_{\begin{array}{c} r_{1}>\ldots >r_{1}\\ z_{1}\in c_{1},\ldots ,z_{2}\in c_{1} \end{array}}{\left(F\left(r_{k}\right)\right)}^{n-k}d\mu \left(r_{1}z_{1}\right)\ldots d\mu \left(r_{k}z_{k}\right)=\\ \frac{n!}{\left(n-k\right)!}\int_{r_{1}>\ldots >r_{1}}{\left(F\left(r_{k}\right)\right)}^{n-k}dFc_{1}\left(r_{1}\right)\ldots dFc_{k}\left(r_{k}\right)=\\ n\left(\begin{array}{c} n-1\\ k-1 \end{array}\right)\int_{r_{1}\ldots p_{1}\;1>r_{1}}{\left(F\left(r_{1}\right)\right)}^{n-k}dFc_{1}\left(r_{1}\right)\ldots dFc_{k}\left(r_{k}\right)=\\ n\left(\begin{array}{c} n-1\\ k-1 \end{array}\right)\int_{0}^{\infty }{\left(F\left(r_{k}\right)\right)}^{n-k}dFc_{k}\left(r_{k}\right)\int_{r_{k}}^{\infty }\ldots \int_{r_{1}}^{\infty }dFc_{1}\left(r_{1}\right)\\ \ldots dFc_{k-1}\left(r_{k-1}\right)=n\left(\begin{array}{c} n-1\\ k-1 \end{array}\right)\int_{0}^{\infty }{\left(F\left(r_{k}\right)\right)}^{n\cdot k}\\ \left(1-Fc_{1}\left(r_{k}\right)\right)\ldots \left(1-Fc_{k-1}\left(r_{k}\right)\right)dFc_{1}\left(r_{k}\right)=\\ n\left(\begin{array}{c} n-1\\ k-1 \end{array}\right)\left(\int_{0}^{r}\left(\ldots \right)+\int^{\infty }\left(\ldots \right)\right)\sim n\left(\begin{array}{c} n-1\\ k-1 \end{array}\right)\int_{0}^{\infty }\left(\ldots \right)= \end{array}$$

(for large *r* uniformly in *n*)

$$\begin{array}{l} n\left(\begin{array}{c} n-1\\ k-1 \end{array}\right)\rho \left(c_{1}\right)\ldots \rho \left(C_{k-1}\right)\int_{r}^{\infty }{\left(1-F\left(r_{k}\right)\right)}^{k-1}{\left(F\left(r_{k}\right)\right)}^{n-k}\\ dFc_{k}\left(r_{k}\right)+\in \to \rho \left(C_{1}\right)\ldots \rho \left(C_{k}\right)+\in , \end{array}$$

as *n*→∞, where we have used Eq. (14) and applied an argument similar to that in Lemma 3, Asymptotically, the probability is factorized, whence the statement □

To prove the convergence we combine in what follows the above lemma and Eq. (13). The idea is that the points with top layer ranks have also small ranks in the radial components. On the other hand, conical extremality of the points with small layer ranks is determined by their, almost independent, spherical components.

Introduce the random variables

$$\begin{array}{r} T_{n}^{\left(0\right)}=0;T_{n}^{\left(k\right)}=\min\{i:\cup_{m-1}^{l}{ℒ}^{\left(n\right)}\left({ℋ}_{n}\right)\cap \{{X_{n}}^{\left|i-1\right|},\\ \ldots ,X_{n}^{\left[n\right]}\}=0\},\;k=1,\ldots ,n, \end{array}$$

which count the *Xi* ‘s in the norm-decreasing order until the first *k* layers having been filled. Clearly, $V_{n}^{\left(k\right)}\leq T_{n}^{\left(k\right)}\leq T_{n}^{\left(k+1\right)}$ Denote *S^∞^* the product of infinitely many spheres, and set

$$\begin{array}{r} {\hat{T}}^{\left(0\right)}\left(z\right)=0;{\hat{T}}^{\left(k\right)}\left(z\right)=\min\{j:\left(S_{+}\cap \left\{z_{1},\ldots ,z_{j}\right\}\right)=k\},\\ {\hat{V}}^{\left(k\right)}\left(z\right)=\#\left\{j:{\hat{T}}^{\left(k-1\right)}\left(z\right)<j⩽{\hat{T}}^{\left(k\right)}{\left(z\right)}_{r}z_{j}\in S\backslash S_{-}\right\}, \end{array}$$

where z − (*z*_1_ *z*_2_,…)∈ *S^∞^* and inf 0 = ∝, For *i* ≥ *j* the set

$$\left\{z\in S^{\infty }:{\hat{V}}^{\left(k\right)}\left(z\right)=j,{\hat{T}}^{\left(k\right)}\left(z\right)=i\right\}$$

is a finite-dimensional cylinder in *S^∝^*.

Denote Z − (Z^[1]^, Z^[2]^,…) the sequence with *iid* components distributed according to *ρ*, $T^{\left(k\right)}={\hat{T}}^{\left(k\right)}\left(Z\right)$, $V^{\left(k\right)}={\hat{V}}^{\left(k\right)}\left(Z\right)$. It follows from the definition and condition (iii) of Sec. 2 that *T*^(^*^k^*^)^, *k* = 1, 2, …is a strictly increasing sequence of finite stopping times with respect to Z^[1]^, Z^[2]^, …

**Theorem 2.***Assume* (i)-(iv), *α* = 0, *and ρ*(*∂S_+_* ∪ *∂S*_−_) = 0. *Then for any k* = 1, 2,…

$$\left(V_{n}^{\left(1\right)},T_{n}^{\left(1\right)},\ldots ,V_{n}^{k},T_{n}^{k}\right)\overset{d}{\to }\left(V^{\left(1\right)},T^{\left(1\right)},\ldots ,V^{\left(k\right)},T^{\left(k\right)}\right).$$

*Proof.* Fix integers *v*_1_,…*v_k_*; *t*_1_,…, *t*_k_ = *t* satisfying 0 < *t*_1_ <… < *f_k_* and 0 ⩽ *v_i_* ⩽ *t_i_*− *t_i_*_−1_, for *i* = 1,…*k.* We need to prove that

$$\begin{array}{c} \underset{n\to \infty }{\lim}\;P\left\{V_{n}^{\left(i\right)}=v_{i},T_{n}^{\left(i\right)}=t_{i};\;i⩽k\right\}=\\ P\left\{V^{\left(i\right)}=v_{i},T^{\left(i\right)}=t_{i};i⩽k\right\}. \end{array}$$

We endow *S^t^* with the product measure *ρ^r^* and the Euclidean metric. Define

$$\begin{array}{r} D=\partial S_{+}\cup \partial S_{-},D=\{\left(z_{1},\ldots ,z_{1}\right)\in S^{\prime }:\\ \left\{z_{1},\ldots ,z_{1}\right\}\cap D=0\}\\ A=\{\left(z_{1},\ldots ,z_{1}\right)\in S^{1}:{\hat{V}}^{\left(i\right)}\left(z_{1},\ldots ,z_{1}\right)=\\ v_{1},{\hat{T}}^{\left(i\right)}\left(z_{1},\ldots ,z_{1}\right)=t_{i};i\leq k\}. \end{array}$$

The definition of A is correct due to the cylindrical property. It is easy to see that D is compact, A\D is open and, by the assumption, $\rho^{\prime }\left(D\right)=0$. It follows, *ρ*^1^*(A)* = *ρ*^1^(*A*/*D*). For any *S* there exists *As* with the properties:

$$\begin{array}{l} A_{\delta }\subset A\backslash D,\rho \left(A\right)-\rho \left(A_{\delta }\right)<\delta ,\;\mathrm{dist}\left(A_{\delta },D\right)>0,\\ \rho^{1}\left(\partial A_{\delta }\right)=0. \end{array}$$ (16)

To prove this, take $C_{\theta }=\{a\in A\backslash D\;;\mathrm{dist}\;(a,\partial \left(A\backslash D\right);\mathrm{dist}\;\left(a,\partial \left(A\backslash D\right)\right)>\theta \}$, then $C_{\theta }$ is an open set, increasing to A\D as *θ* ↓ 0. We have $\rho^{\prime }\left((A\backslash D)\backslash C_{\theta }\right)<\delta$ for sufficiently small *θ*. On the other hand, $\partial C_{\theta }\subset \{a\in S^{l}:\mathrm{dist}\left(a,\partial \left(A\backslash D\right)\right)=\theta \}$, these sets being disjoint for different *θ*, Hence the set of the values of *θ* with $\rho^{l}\left(\partial C_{\theta }\right)>0$ is at most countable. Select an appropriate *θ* and set $A_{\delta }-C_{\theta }$.

We derive from Eq. (16) with the help of some topological considerations that for sufficiently small *ϵ*

$$\underset{i\leq 1}{\cup }\;\left(z_{l}+B_{\in }\right)\;\cap \;cone\left(D\right)=\theta \;\left(z_{1},\ldots ,z_{1}\right)\in A_{\delta }.$$ (17)

Assume now that the compound event

$$\left(Z_{n}^{\left[1\right]},\ldots ,Z_{n}^{\left|r\right|}\right)\in \;A_{\delta };\;\in R_{n}^{\left[l\right]}>R_{n}^{\left[i+l\right]}\;i=1,\ldots ,k$$ (18)

occurs. We show next that in this case

$$T_{n}^{\left(r\right)}=l_{i},\;V_{n}^{\left(f\right)}=v_{i}\;i=1,\ldots ,k.$$ (19)

Let *Q* be an clement of the finite algebra of spherical sets generated by S_+_ and S_−_ The following equivalence holds:

$$X_{n}^{\left[i\right]}-X_{n}^{\left[j\right]}\in cone\left(Q\right)\Leftrightarrow Z_{n}^{\left|i\right|}\in Q\;for\;1\leq i\leq 1,i<j\leq n.$$ (20)

Indeed, note first that *∂Q* ⊂ D. By (17) $Z_{n}^{\left[l\right]}\in Q$ implies $Z_{n}^{\left[i\right]}+B_{c}\subset \;\mathrm{cone}\;\left(Q\right)$. From Eq. (18) we have also $X_{n}^{\left[i\right]}+B_{k_{n}^{\left[i\right]}}\subset \mathrm{cone}\left(Q\right)$. But $-X_{n}^{\left[i\right]}\in B_{k_{n}^{\left[j\right]}}$ thus $X_{n}^{\left[i\right]}-X_{n}^{\left[i\right]}\in \mathrm{cone}\left(Q\right)$. Use *Q^C^*instead of *Q* to prove the reverse implication.

The definition of *A* and Eq. (18) yield A, *i* = 1, …, *k*. Setting *Q = S_+_* in Eq. (20) we have $X_{n}^{\left[t_{l}\right]}-X_{n}^{\left[j\right]}\in K,\;t_{i}<j\leq n$. Therefore,

$$\{X_{n}^{\left[l_{r}+1\right]},\ldots ,X_{n}^{\left[n\right]}\}\cap \left(\overset{k}{\underset{m\;=\;1}{\cup }}{ℒ}^{\left(m\right)}\left({ℋ}_{n}\right)\right)=\theta$$

Let t*_i_*_−1_ *< j <t_i_* and $Z_{n}^{\left[j\right]}\in S$. Setting *Q* − *S.* in Eq. (20), we have $\{X_{n}^{\left|j-1\right|},\ldots ,\;X_{n}^{\left[n\right]}\}\subset \left(X_{n}^{\left[j\right]}+K\right)$. Setting *Q* = S_+_ we have further $\{X_{n}^{\left|f_{1}\right|},\;X_{n}^{\left|f_{2}\right|},\ldots X_{n}^{\left|f_{l-1}\right|}\}\;\in \left(X_{n}^{\left[j\right]}+K\right)$. That is, $X_{n}^{\left[j\right]}\notin \cup_{m\;\leq k}{ℒ}^{\left(m\right)}\left({ℋ}_{n}\right)$.

Let t*_i_*_−1_ *< j <t_i_* and $Z_{n}^{\left[j\right]}\in S_{0}$. Substituting *Q = S*_0_ into Eq. (20) we get $X_{n}^{\left[j\right]}-X_{n}^{\left[p\right]}\in \;\mathrm{cone}\;\left(S_{0}\right)$, *j* + 1 ≤ *p* ≤ *n*, together with *S*_0_ − *S*_0_ and *S*_0_ ∩ *S*_+_. = 0 this yields $\{X_{n}^{\left|j+1\right|},\ldots ,X_{n}^{\left[n\right]}\}\cap \left(K+X_{n}^{\left[j\right]}\right)=0$. For *Q* = S\S_+_ and *p* ∈{1, …; *j*−l}\{*t*_1_, *t*_2_,…t*_i_*_−1_} we have $X_{n}^{\left[p\right]}\notin \left(K+X_{n}^{\left[n\right]}\right)$. Similarly, for p ∈ {*t*_1_, *t*_2_,…, t*_i_*_–1_ } we have

$$\{X_{n}^{\left[r_{1}\right]},X_{n}^{\left[r_{2}\right]},\ldots ,X_{n}^{\left[r_{i-1}\right]}\}\subset X_{n}^{\left[j\right]}+K.$$

Thus in this case $X_{n}^{\left[j\right]}\in {ℒ}^{\left(i-1\right)}\left({ℋ}_{n}\right)$.

In the same manner, $Z_{n}^{\left|l_{r}\right|}\in S_{+}\backslash S$, implies $X_{n}^{\left[r_{l}\right]}\in {ℒ}^{\left(i-1\right)}({ℋ}_{n})$.

Summarizing, if Eq. (18) holds then ${ℒ}^{\left(i\right)}\left({ℋ}_{n}\right)=\{X_{n}^{\left[l_{1}\right]}:t_{i-1}<j\leq t_{j},\;Z_{n}^{\left[j\right]}\in S,\backslash S_{-}\}$, whence Eq. (19).

Now from Eq. (13) and Lemma 5 (recall that *t − t_k_*)

$$\begin{array}{c} P\{\left(Z_{n}^{\left[1\right]},\ldots ,Z_{n}^{\left|r_{k}\right|}\right)\in A_{\delta },\;\in R_{n}^{\left|i\right|}>R_{n}^{\left[i+1\right]};\\ i=1,\ldots ,l_{k}\}\to \rho^{r_{k}}\left(A_{\delta }\right) \end{array}$$

Recalling the definitions of A and $A_{\theta }$ we get

$$\begin{array}{c} \underset{n\to \infty }{\lim\;\inf}\;P\left\{V_{n}^{\left(i\right)}=v_{i},\;T_{n}^{\left(i\right)}=t_{i};\;i⩽k\right\}>\\ P\left\{V^{\left(i\right)}=\;v_{i},\;T^{\left(i\right)}=t_{i};\;i⩽k\right\}-\delta . \end{array}$$

Take *δ = δ*(*v*_1_…, *v_k_*; *t*_1_,…, *t_k_*) with ∑*δ*(*v*_1_,…, *v_k_*; *t*_1_,…, *t_k_) = β* and choose a diagonal subsequence of the values of *n* to get the convergence of the probabilities in the left-hand side. Recalling that probabilities sum to one, we derive Eq. (15) by setting β→0 □

Convergence in mean does not require additional restrictions, as shown next.

**Theorem 3**. *Under the assumptions of Theorem 2*

$$EV_{n}^{\left(k\right)}\to EV^{\left(k\right)},\;\;n\to \infty$$

*Proof.* It is sufficient to consider only the case *k* = 1. Denote by *I_n_*^[^*^i^*^]^ and *I*^[^*^i^*^]^ the indicator functionsof the events $\{X_{n}^{\left[i\right]}\in {ℒ}^{\left(1\right)}\left({ℋ}_{n}\right)\}$ and $\{Z^{\left[i\right]}\in S\backslash S_{-},\;i\leq T^{\left[1\right]}\}$ respectively. Clearly,

$$V_{n}^{\left(1\right)}=l_{n}^{\left[1\right]}+\ldots l_{n}^{\left[n\right]},\;V^{\left(1\right)}=I^{\left[1\right]}+I^{\left[2\right]}+\ldots$$

By an argument similar to that used in Theorem 2 we show that

$$\left(1_{n}^{\left[1\right]},\ldots ,I_{n}^{\left[m\right]}\right)\overset{d}{\to }\left(I^{\left[1\right]},\ldots ,I^{\left[m\right]}\right)\;m=1,2,\ldots$$ (21)

Choose *γ_n_* satisfying *np_n_→*λ, where $\lambda >0,\;p_{n}=1-\mu \left(B_{\gamma_{n}}\right)$. The random variable $N=\#\left(\{X_{1},\ldots ,X_{n}\}\cap B_{\gamma_{n}}^{c}\right)$ has binomial distribution with parameters (*n*, *P_n_).* By Eq. (6),

$$E\left({I_{n}}^{\left[N+1\right]}+\ldots +I_{n}^{\left[n\right]}\right)=E\left({ℒ}^{\left(1\right)}\left({ℋ}_{n}\right)\cap B_{r_{n}}\right)\leq {e-}^{\lambda t}\tau^{1}.$$ (22)

Fix *m* and write the expectation as

$$\begin{array}{c} EV_{n}^{\left(1\right)}=E\left(I_{n}^{\left[1\right]}+\ldots I_{n}^{\left[n\right]}\right)=E\left(I_{n}^{\left[1\right]}+\ldots I_{n}^{\left[m\right]}\right)+\\ E\left(I_{n}^{\left[m+1\right]}+\ldots I_{n}^{\left[N\right]}\right)1_{\{N\geq m}\}+E\left(I_{n}^{\left[N+1\right]}+\ldots I_{n}^{\left[n\right]}\right)1_{\left\{N\geq m\right\}}+\\ E\left(I_{n}^{\left[m+1\right]}+\ldots I_{n}^{\left[N\right]}\right)1_{\left\{N<m\right\}}. \end{array}$$

The first term converges by Eq. (21):

$E\left(I_{n}^{\left|l\right|}+\ldots I_{n}^{\left|m\right|}\right)\to E\left(I^{\left|l\right|}+\ldots I^{\left|m\right|}\right)$. The first and the third terms arc estimated by Eq. (22) as

$$\begin{array}{l} E\left(I_{n}^{\left[N+1\right]}+\ldots I_{n}^{\left[n\right]}\right)1_{\left\{N\geq m\right\}}+E\left(I_{n}^{\left[N+1\right]}+\ldots I_{n}^{\left[n\right]}\right)1_{\left\{N<m\right\}}\\ \leq E\left(I_{n}^{\left[n+1\right]}+\ldots I_{n}^{\left[n\right]}\right)\leq e^{\Lambda \tau }\tau^{1}. \end{array}$$

Since *N* is binomially distributed, we have for the second term

$$\begin{array}{l} E\left(I_{n}^{\left[N+1\right]}+\ldots I_{n}^{\left[n\right]}\right)1_{\left\{N\geq m\right\}}<E\left(N1_{\left\{N\geq m\right\}}\right)\to \\ \lambda P\left\{N_{\Lambda }\geq m-1\right\},n\to \propto \end{array}$$

where *N_λ_* is a Poisson random variable with parameter *λ*. Selecting *λ* and then *m* sufficiently large, we prove $\sup_{n\to x}EV_{n}^{\left(k\right)}\leq EV^{\left(k\right)}$.

The inverse inequality involving lim inf follows from the convergence in distribution □

It is not hard to find the limiting distributions of the $V_{n}^{\left(k\right)}$’S. Note first that

$$\left(V^{\left(1\right)},T^{\left(1\right)}\right),\left(V^{\left(2\right)},T^{\left(2\right)}-T^{\left(1\right)}\right),\ldots$$

are all *iid*, therefore it is sufficient to consider only the first pair. Clearly *T*^(1)^ is geometrically distributed with parameter *ρ*(*S_+_*). The probability law of *V*^(1)^ is found from the following scheme: throw down the *iid* points *Z*^[1]^ *Z*^[2]^,… in S according to the probability law *ρ* until the first point falls into *S_+_*, then count all the points falling into *S\S*_−_ To make this precise denote

$$p_{0}=\rho \left(S_{0}\right),p_{2}=\rho \left(S_{+}\right),p_{-}=\rho \left(S_{-}\right),p_{+}=\rho \left(S_{+}\right)$$

(thus *p*_0_ *+ p +p_+_− p_−_* = 1). The joint distribution of *V*^(1)^ and *T*^(1)^ is this:

$$\begin{array}{l} P\left\{V^{\left(1\right)}=i,T^{\left(1\right)}-j\right\}-P\{\left\{Z^{\left[1\right]},\ldots ,Z^{\left[j-1\right]}\right\}\cap S_{+}=0.\\ Z^{\left[j\right]}\in S_{+},\#\left(\left\{Z^{\left[1\right]},\ldots ,Z^{\left[j\right]}\right\}\cap \left(S\backslash S_{-}\right)\right)=i\}=\\ P\{\left\{Z^{\left[1\right]},\ldots ,Z^{\left[j\right]}\right\}\cap S_{+}=0,\\ Z^{\left[j\right]}\in S_{+}\backslash S,\#\left(\left\{Z^{\left[1\right]},\ldots ,Z^{\left[j-1\right]}\right\}\cap \left(S\backslash S_{-}\right)\right)-i-1\}+\\ P\{\left\{Z^{\left[1\right]},\ldots ,Z^{\left[j-1\right]}\right\}\cap S_{+}=0,Z^{\left[j\right]}\in S_{\pm },\\ \#\left(\left\{Z^{\left[1\right]},\ldots ,Z^{\left[j-1\right]}\right\}\cap \left(S\backslash S_{-}\right)\right)-i\}=\\ P\{\#\left(\left\{Z^{\left[1\right]},\ldots ,Z^{\left[j-1\right]}\right\}\cap S_{0}\right)=i-1,\\ \#\left(\left\{Z^{\left[1\right]},\ldots ,Z^{\left[j-1\right]}\right\}\cap \left(S_{-}\backslash S_{+}\right)\right)=j-i\}\times \\ P\left\{Z^{\left[j\right]}\in S_{+}\backslash S_{-}\right\}+P\{\#\left(\left\{Z^{\left[1\right]},\ldots ,Z^{\left[j-1\right]}\right\}\cap S_{0}\right)=i,\\ \#\left(\left\{Z^{\left[1\right]},\ldots ,Z^{\left[j-1\right]}\right\}\cap \left(S_{-}\backslash S_{+}\right)\right)-j-i-1\}P\left\{Z^{\left[j\right]}\in S_{z}\right\}=\\ \left(\begin{array}{c} j-1\\ i-1 \end{array}\right)p_{0}^{i-1}{\left(p_{+}-p_{\pm }\right)}^{j-i}\left(p_{+}-p_{\pm }\right)+\left(\begin{array}{c} j-1\\ i \end{array}\right)p_{0}^{1}{\left(p_{-}-p_{\pm }\right)}^{j-1}p_{\pm }, \end{array}$$

where *i* ≥ 0, *j* ≥ 1 and *j* ≥ *i* Summing over *j* we arrive at the limiting distribution of points in the *k*th layer:

$$\begin{array}{c} P\left\{V^{\left(k\right)}=0\right\}=\frac{p_{\pm }}{1-p+p_{\pm }},\\ P\left\{V^{\left(k\right)}=i\right\}=\frac{p_{0}^{i-1}\left(1-p_{-}\right)p_{+}}{{\left(1-p_{-}+p_{\pm }\right)}^{i+1}}i=1,2\ldots . \end{array}$$

If the cone satisfies *K ∩ −K* = 0 (or, more generally p(*S_+_* ∩ *S*_−_) = 0) then p= −0 and *V*^(^*^k^*^)^ is geometrically distributed. A little additional work is needed to find the expectation:

$$EV^{\left(k\right)}=\frac{1-p}{p_{+}}.$$ (23)

**Example.** Assume that the radial tail is slowly varying and *ρ* is the uniform spherical measure.

For $K=R_{+}^{d}$ we have *p*_+_, = *p*_−_. = 2*^d^*, and Eq. (23) yield $EV_{n}^{\left(k\right)}\to 2^{d}-1$. In particular, the mean number of Pareto points in two dimensions converges to 3.

Taking the complement to the negative orthant. we have *p*_+_ = *p*_= 1−2^−d^ and $EV_{n}^{\left(k\right)}\to {\left(2^{d}-1\right)}^{-1}$. In two dimensions. the probability that the sample has the double maximum tends to 1/3.

Appearing of inverse numbers in the above example is a general phenomenon. We write further $V_{n}^{\left(k\right)}\left(k\right)$ to emphasize the dependence on the cone.

**Theorem 4.** Under the assumptions of Theorem 2

$$\underset{n\to \infty }{\lim}EV_{n}^{\left(k\right)}\left(K\right)EV_{n}^{\left(k\right)}\left(-K\right)=1,$$

*provided one of the numbers p*_+_ or 1−*p*_−_ *is positive.*

*Proof.* This follows from Theorem 3, Eq. (23), and the formulas *p*_−_(−*K*^c^) = 1−*p*,(K), *p*_+_(−*K*^c^) = 1−*p*−(*K*) □

**Remark.** Given a binary relation, say ℛ on a sampling space, and a random sample ${ℋ}_{n}$, there are two natural ways to define the “*k*th extremes” *of*, $K_{n}$ *:* (1) sample elements *X_i_* which are in ℛ with all other sample elements with the exceptbn of some *k*−1 points; or (2) the elements *X_i_* such that there are exactly *k* − 1 sample points which are in the relation with *X,.* In the theory of partially ordered sets extremes (*k* − 1) of the first type are called the greatest points, of the second type—maximal [4]. This is best illustrated by the natural partial order of **R***^d^*: total maximum is the greatest point, while Pareto set consists of maximal points. If the binary relation ℛ is generated by a cone *K*, as mentioned in Introduction, then the *K*-extremes are maximal points, while the *−K*^c^ extremes are the greatest points w.r.t. ℛ. Baryshnikov [3] has proved that the asymptotic upper bound for the product of expectations of the numbers of the extremes of both types is at most 1, for any fixed ℛ and *k.* Theorem 4 shows that this bound is sharp.

**Remark.** Normal multivariate distributions can be viewed as the case of fast decreasing radial tails, *a* − ∝. The mean number of conical extremes demonstrates typically the following behavior: for any *k*, $EV_{n}^{k}$ infinitely grows if *K* is contained in a half-space, and tends to zero if *K* contains a half-space [10, 11].
